# The complete plastome of a cultivar of *Lannea coromandelica*

**DOI:** 10.1080/23802359.2021.1998803

**Published:** 2021-11-11

**Authors:** Qi-Liang Zhou, Zi-Hao Tan, Hong-Xin Wang, Da-Juan Chen, Xiu-Rong Ke, Zhi-Xin Zhu, Hua-Feng Wang

**Affiliations:** Hainan Key Laboratory for Sustainable Utilization of Tropical Bioresources, College of Tropical Crops, Hainan University, Haikou, China

**Keywords:** *Lannea coromandelica*, Anacardiaceae, plastome, genome structure

## Abstract

*Lannea coromandelica* (Houtt.) Merr. is a deciduous tree in the family Anacardiaceae, which grows in lowland and hill forests; 100–1800 m. SW Guangdong, S Guangxi, S Yunnan [Bhutan, India, Myanmar, Nepal, Sri Lanka; cultivated elsewhere in continental SE Asia, such as in Cambodia, Laos, Malaysia, Thailand, Vietnam, where it is probably naturalized]. The length of the complete plastome is 162,460 bp, including 130 genes consisting of 85 protein-coding genes, 37 tRNA genes and 8 rRNA genes. The assembled plastome has the typical structure and gene content of angiosperms plastome, which includes two inverted repeats (IRs) regions of 26,877 bp, a large single copy (LSC) region of 89,599 bp and a small single-copy (SSC) region of 19,107 bp. The total G/C content in the plastome of *L. coromandelica* is 37.7%. The complete plastome sequence of *L. coromandelica* will provide contributions to the conservation genetics of this species as well as to phylogenetic studies in Anacardiaceae.

## Introduction

*Lannea coromandelica* (Houtt.) Merr. is a deciduous tree, which can grow up to 5–10 m tall, with gray-white bark, thick, branchlets densely covered with rust-colored stellate hairs. Leaves are odd-pinnate often clustered at branchlets, 10 − 33 cm long, with (5-)7-9(-11) pairs of leaflets, 2 or 5 pairs of rachis and cylindrical petioles, sparsely covered with rust-colored stellate hairs. The flowers are small, yellow or purplish, arranged in terminal branched or unbranched racemes. Drupes are ovate, slightly compressed, purplish-red when ripe, 6–10 mm long, ca. 0.5 mm wide, glabrous. Tannin extracts can be extracted from the bark of *L. coromandelica*, and fishnets can be stained with extracts. The stem bark fiber can be woven into coarse cloth. Seeds can be pressed into oil. Bark can be used as medicine, with free radical scavenging, anti-tumor, inhibition of cancer cells and other effects. Therefore, we reported the complete plastome of *L. coromandelica* (Houtt.) Merr. in this study, which is expected to improve the quality of the relevant collection, medical application and phylogenetic investigation of Anacardiaceae.

In this study, a fruiting cultivar of *L. coromandelica* (Houtt.) Merr. was sampled from Qionghai city in Hainan (109.24°E, 18.55°N). A voucher specimen (voucher code: X F Zhang, A245, HUTB) and its DNA were deposited in the Herbarium of Hainan University, Hainan province, China (code of herbarium: HUTB).

The experiment was carried out as reported in Zhu et al. (2018). Clean data were assembled using the plastid of *Rhus typhina* NC 046837.1 as a reference with MITObim v1.8 (Hahn et al. 2013). The plastome was annotated against the plastome of *Rhus typhina* NC 046837.1 by using Geneious R8.0.2 (Biomatters Ltd, Auckland, New Zealand) with subsequent correction of the annotation conducted with DOGMA (Wyman et al. 2004).

Our results show that the plastome of *L. coromandelica*, has a typical quadripartite structure of angiosperms and a full length of 162,460 bp, consisting of 26,877 base pairs for two Inverted Repeats (IRs), 89,599 base pairs for the Large Single Copy (LSC) region and 19,107 base pairs for the Small Single-Copy (SSC) region. The plastome consists of 130 genes, consisting of 85 protein-coding genes (six of which are duplicated in the IR), 37 tRNA genes (eight of which are duplicated in the IR) and 8 rRNA genes (5s rRNA, 4.5s rRNA, 23s rRNA and 16s rRNA) (four of which are duplicated in the IR). The total G/C content of the plastome of *L. coromandelica* was 37.7%, and the G/C content of the LSC, SSC, and IR regions were 35.8, 32.2, and 42.8%, respectively.

**Figure 1.**
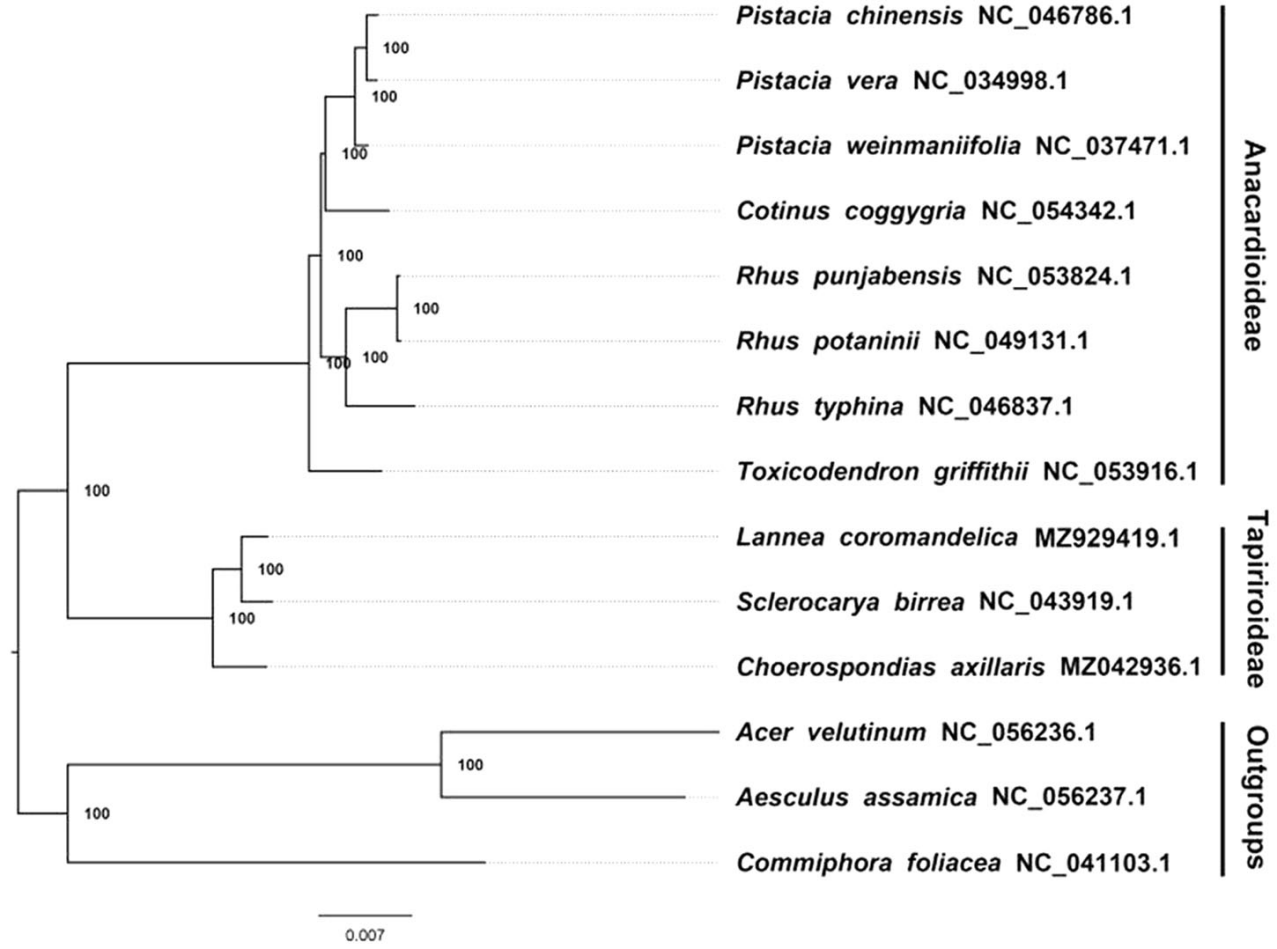
The maximum likelihood phylogeny recovered from 14 complete plastome sequences using RAxML. Accession numbers: *Grielum grandiflorum* (GenBank accession number, MZ929419, this study), *Acer velutinum* NC 056236 1, *Aesculus assamica* NC 056237 1, *Choerospondias axillaris* MZ042936 1, *Commiphora foliacea* NC 041103 1, *Cotinus coggygria* NC 054342 1, *Pistacia chinensis* NC 046786 1, *Pistacia vera* NC 034998 1, *Pistacia weinmaniifolia* NC 037471 1, *Rhus punjabensis* NC 053824 1, *Rhus potaninii* NC 049131 1, *Rhus typhina* NC 046837 1, *Sclerocarya birrea* NC 043919 1, *Toxicodendron griffithii* NC 053916 1.

We constructed a maximum likelihood (ML) phylogeny of 13 published complete plastomes of Anacardiaceae using the online CIPRES portal (http://www.phylo.org/portal2/login!input.action) with 1,000 bootstraps (Stamatakis 2006). *Acer velutinum* NC 056236.1, *Aesculus assamica* NC 056237.1 and *Commiphora foliacea* NC 041103.1 were used as outgroups. By inferring the phylogenetic relationship based on the existing data and related taxa, we find that *Lannea coromandelica* is closer to *Sclerocarya birrea* than other species in this study (Figure 1).

The current data and investigation show that most nodes of the plastome ML trees are fully supported. At present, the plastid sequence of common Anacardiaceae plants has gradually increased. This is of great significance for promoting the related protection and phylogenetic research of Anacardiaceae plants, and for deepening the understanding of these plants.

## Data Availability

The genome sequence data supporting the results of this study are publicly available in NCBI GenBank (https://www.ncbi.nlm.nih.gov/) with registration number MZ929419. The associated BioProject, SRA, and Bio-Sample numbers are PRJNA748537, SRR15651128 and SAMN20858431, respectively.
